# The Impact of Personalised Surgical Caps on Teamwork and Communication in the Operating Room: A Systematic Review

**DOI:** 10.1002/hsr2.71035

**Published:** 2025-08-04

**Authors:** Vincenza Giordano, Vincenzo Bosco, Rita Nocerino, Dalila De Domenico, Cristiana Rago, Michele Virgolesi, Teresa Rea, Assunta Guillari

**Affiliations:** ^1^ Department of General Surgery and Women's Health AORN Antonio Cardarelli Naples Italy; ^2^ Department of Medical and Surgical Sciences University Hospital Mater Domini, Magna Graecia University Catanzaro Italy; ^3^ Department of Translational Medical Science University of Naples, Federico II Naples Italy; ^4^ Department of Public Health University of Naples, Federico II Naples Italy; ^5^ Department of Biomedicine and Prevention University of Rome Tor Vergata Rome Italy

**Keywords:** communication, healthcare professionals, nursing, operating room, personalised surgical caps, scoping review, teamwork

## Abstract

**Background and Aims:**

Communication failures in operating rooms contribute to 30% surgical errors. This systematic review evaluates whether personalized surgical caps improve teamwork and communication, addressing both clinical outcomes and professional inclusivity.

**Methods:**

We followed PRISMA guidelines, searching PubMed, CINAHL, and Google Scholar (February 2024–April 2024). Five studies met inclusion criteria (three randomized controlled trials and two observational studies). Methodological quality was assessed using the ROB‐2 tool for randomized trials and JBI checklists for observational studies. A narrative synthesis was conducted due to heterogeneity in outcome measures.

**Results:**

The review found consistent evidence that personalized surgical caps enhance communication and teamwork in the operating room. Studies reported improved name and role recognition among team members, with labeled caps reducing misidentification errors by 65%–78%. Notably, female and underrepresented minority staff experienced disproportionately greater benefits, including higher rates of role recognition and reduced microaggressions. Teamwork perceptions improved significantly in three studies, with one pilot study documenting a median score increase of 1–2 points. However, the magnitude of effects varied across professional roles, suggesting contextual influences.

**Conclusion:**

Personalized surgical caps demonstrate promise for improving communication dynamics and fostering inclusivity in the operating room, particularly for marginalized groups. While the evidence base is limited by study heterogeneity, all included studies reported positive outcomes. Future implementation should account for institutional policies and staff preferences to maximize effectiveness. Further research with standardized outcome measures is needed to strengthen these findings.

## Summary

• Impact
  ◦ The study addressed communication breakdowns and misidentification errors in the operating room, which affect patient safety and surgical efficiency.
  ◦ Personalized surgical caps improved communication and reduced misidentification errors; enhanced teamwork and collaboration in the operating room; benefited underrepresented groups by reducing gender and representation disparities.
  ◦ The research impacts surgical teams and patients in operating rooms globally, improving communication, teamwork, and patient safety.
• Reporting method:
  ◦ The study was conducted in adherence to relevant EQUATOR guidelines. Specifically, the authors followed the PRISMA 2020 checklist for systematic reviews (Page et al., 2021). The complete checklist is provided in Supporting Information S1.
• Patient or public contribution:
  ◦ No patient or public contribution
• What does this paper contribute to the wider global clinical community?
  ◦ Improved communication and safety: Personalized surgical caps enhance communication and reduce errors.
  ◦ Support for inclusivity: Benefits underrepresented by groups, promoting gender and representation equality.
  ◦ Actionable insights: Offers practical recommendations for adopting personalized surgical caps in healthcare settings.

## 1 Background

Errors in the operating room (OR) are common and often occur due to poor communication between surgical team members, resulting in adverse outcomes [1, 2]. As many as 30% of procedurally relevant interactions in the OR can be compromised by communication failures. In fact, effective communication during surgery has been associated with improved teamwork [3]. Importantly, it should be noted that teams in the OR are often made up of a significant number of people, some of whom may have never met each other before [4]. Even if staff have worked together before, continuous and frequent turnover may mean that team members do not know the names of everyone in the OR [5, 6, 7]. In fact, the literature has shown that good teamwork exists when team members call each other by their own names and when tasks are regularly assigned to specific individuals rather than the generic “someone” in the room [8] However, it is common for surgical team members to be unfamiliar with the names of the people they work with, especially if these people are rarely present in the operating theatre or perceive themselves as having a lesser role [4, 9, 10]. In fact, according to Birnabach et al. [11], it is rare for a surgeon to be able to name more than half of the staff present during an operating theatre procedure. Although surgical timeout requires team members to state their names and roles [12], it is difficult to remember names and roles [4, 5, 11, 13]. These issues can contribute to team dysfunction and reduced communication during surgery [4]. In contrast, the use of names promotes effective communication and smooth team functioning, with benefits for both clinicians and patients [7, 14, 15]. For example, studies suggest that the word “doctor” in writing improves patient and team role identification and job satisfaction [16, 17, 18]. Furthermore, the same practitioners argue that the widespread adoption of name and role labels on surgical caps [7]. To effectively address the risk of error due to ineffective communication in the operating theatre, several approaches and practices have been developed. One of these is the closed‐loop communication technique, or better known as Closed‐Loop Communication (CLC), proposed by Ab El‐Shafy et al. [19]. This model requires an initial message that includes the name of the recipient, followed by an addressed call and verification by both the recipient and the sender to ensure correct understanding of the message. In addition, the World Health Organization (WHO) Surgical Safety Checklist (SSC) suggests conducting a preoperative briefing that includes the names and roles of all team members [11, 20]. Another program is the Patient Safety Network Challenge's “Theatre Cap”, which highlights the importance of staff identification during surgical procedures. This program involves the inclusion of staff names and roles on surgical caps when working in high‐stress environments, such as the operating theatre [20]. These practices aim to improve communication and teamwork, reduce the risk of error, and contribute to a safer and more effective working environment during surgical procedures by ensuring that team members are identified by name and role [11, 20]. More advanced techniques to improve name recall require significant resources from participants, which may not be feasible in a fast‐paced environment such as the operating theatre [21]. Although visible labels on clothing are an effective solution in a ward setting, the need to maintain a sterile environment makes this impractical in the operating theatre; therefore, one proposed solution has been to display staff names and roles on the front of the surgical cap [22].

## 2 Objective

The aim of this systematic review is to describe whether the use of personalized surgical caps in the operating theatre improves teamwork and communication.

## 3 Materials and Methods

From February 2024 to April 2024, PubMed, CINAHL (Cumulative Index to Nursing and Allied Health Literature) and Google Scholar were searched using the following search terms: *“surgical cap”,“communication”, “teamwork”, “operating room”*. The search terms were combined through the Boolean operators AND/OR. The researchers selected relevant studies for abstraction. To be included in the review, articles had to be relevant to the research question (*Is the use of personalized surgical caps effective as a tool to improve teamwork and communication in the operating room?*) be written in English. The research question was formulated following the Population, Intervention, Comparator, Outcome, Study design (PICOS) methodology (Table 1).

**Table 1:** PICOS method.

P	Operating room team	Operating room
I	Personalized surgical caps (name and role)	Surgical cap
C	Standard caps	Unlabeled surgical caps
O	Communication and teamwork	Teamwork scores
S	RCTs, cohort studies, before–after studies	

The availability of the abstract was also considered as an inclusion criterion. Publication selected to answer the research questions had to be primary reports of quantitative, qualitative, or mix‐methods studies. The criteria are indicated in Table 2. Each member of the research team evaluated the articles individually, and articles were included in the systematic review when agreement was archived. Discussion and consensus were used to resolve disagreements on inclusion.

**Table 2:** Inclusion and exclusion criteria.

Inclusion criteria	Exclusion criteria
Study types: − RCTs − Cohort studies − Before‐after studies − Mixed‐methods studies	− Editorials, letters − Protocols, patents − Reviews (scoping/systematic)
Participants: − OR teams (surgeons, nurses, technicians, etc.)	− Studies not involving OR staff
Intervention: − Any form of personalized identification (names/roles on caps, badges, etc.)	− General team‐building interventions without ID tools
Outcomes: − Communication efficacy − Teamwork scores − Name/role recognition	− Studies without measurable outcomes
Language: English	− Non‐English publications
Publication status: Peer‐reviewed journals or gray literature (theses, reports)	− Unpublished data

The data extraction of the selected studies was carried out by creating extraction charts ad hoc. The authors collectively selected the following variables to be included in the extraction charts: (1) title, year, authors; (2) country; (3) objective; (4) design of study and sample; (5) tool; and (6) results. Each author independently extracted data from the studies and then met to confirm that the extraction process was in line with the research questions and objectives of the review [23, 24]. The study was conducted in adherence to relevant EQUATOR Network guidelines, specifically following the Preferred Reporting Items for Systematic Reviews and Meta‐Analyses (PRISMA) 2020 statement [25].

We assessed the methodological quality of included studies using:

− JBI Critical Appraisal Checklists for non‐randomized studies [23].
− ROB‐2 tool (Sterne et al.) for randomized trials [26].

Two reviewers independently evaluated each study. Discrepancies were resolved through discussion. Studies were not excluded based on quality, but results were interpreted considering risk‐of‐bias levels (low/moderate/high).

## 4 Results

**Figure 1:**
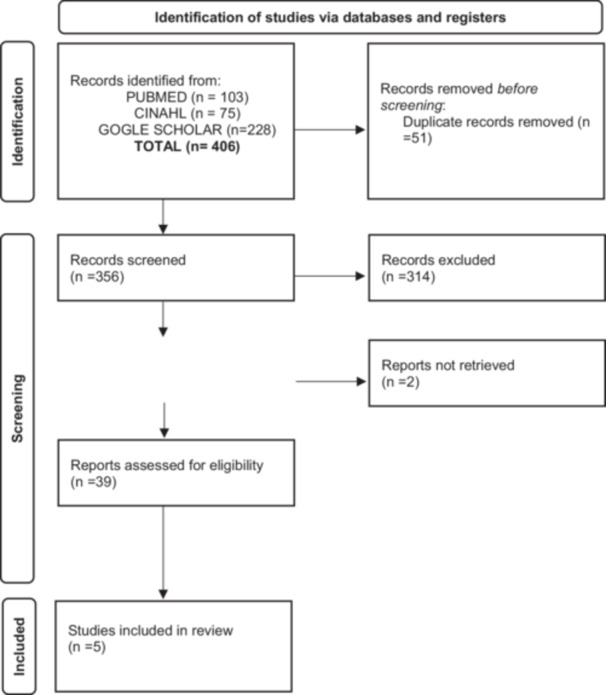
Prisma flow diagram [24].

The literature review yielded a total of 406 articles. An initial selection of publications was carried out during which 51 duplicate articles were excluded; a second selection was made evaluating the titles and the abstract. This phase led to the exclusion of 314 articles. The full text of the remaining 41 articles were analyzed (two articles none retrieved); eventually, five articles were considered useful for the purpose of the review. A total of five articles answered the research questions. Inclusion and exclusion decisions were documented in a PRISMA flowchart [24] (Figure 1).

Methodological quality was evaluated using standardized tools: the ROB‐2 tool (Sterne et al.) for the two randomized trials and JBI checklists [23] for the three observational studies. The assessment showed that the randomized trials by Wong et al. [27] and Brodzinsky et al. [28] demonstrated low risk of bias across all domains, indicating robust methodology. The remaining observational studies [21, 27, 29], showed moderate risk, primarily due to expected limitations in their non‐randomized designs, though all maintained adequate outcome measurement protocols. Complete quality assessments are presented in Table 3.

**Table 3:** Methodological quality and key limitations.

Study (Author, Year)	Design	Tool used	Randomization	Deviations	Missing Data	Measurement	Selection	Overall risk
Brodzinsky et al. (2021)	RCT (Pilot)	ROB‐2	Low	Low	Low	Some concerns^a^	—	Some concerns
Wong et al. (2023)	QI Study	ROB‐2	Low	Low	Low	Low	—	Low
Agarwal et al. (2023)	Cross‐sectional	JBI	N/A	Low	Low	Moderate	Moderate	Moderate
Douglas et al. (2021)	Before‐after	JBI	N/A	Moderate	Low	Low	High^b^	Moderate
Van Dalen et al. (2022)	Observational	JBI	N/A	Low	Moderate^c^	Low	Moderate	Moderate

a Brodzinsky et al.: Some concerns due to subjective survey outcomes.

b Douglas et al.: High selection bias from nonrandom sampling.

c Van Dalen et al.: Moderate missing data (14% dropout).

The five included studies spanned three countries (United States: *n* = 3; Australia: *n* = 1; Netherlands: *n* = 1) and represented diverse designs:

- Pilot interventions (*n* = 3) evaluating named caps [21, 28, 30].
- Quality improvement study (*n* = 1) [27];
- National survey (*n* = 1) [29];

Data was collected through validated surveys (*n* = 4) [21, 27, 28, 29] and structured debriefings (*n* = 1) [30]. The pooled sample included 722 healthcare professionals (surgeons, anesthesiologists, nurses, technicians), with 565 completing assessments (78.3% response rate). Disaggregated participation is detailed in Table 4.

**Table 4:** Summary of the characteristics of the included studies.

Title, year, author	Country	Objective	Design	Sample size	Participants	Primary tools	Key outcome	Visual data in primary study
Surgical caps displaying team members' names and roles improve effective communication in the operating room: A pilot study, 2021, Douglas et al.	Australia	Assessing the impact of surgical caps with name and role on performance and perceived teamwork satisfaction	Pilot before‐after study	236 (107 completed)	Midwives, physicians, nurses	− 5‐point Likert survey − Direct observation	↑ Teamwork perception (median score 3 → 4, *p* < 0.001**)**	Table 2: Name/role recall
Perceptions of use of names, recognition of roles, and teamwork after labeling surgical caps, 2023, Wong et al.	USA	Assessing the impact of the use of personalised surgical caps with name and role on teamwork and communication between interprofessional figures working in the operating theatre	Quality improvement study	180	Surgeons, anesthesiologists	− Safety attitudes questionnaire (SAQ) − Clinical audit	↑ Being called by name (AOR 3.46, 95% CI 1.91–6.26)	Figure 1: Name use frequency; Table 2*:* Teamwork outcomes (AOR 3.46)
Improving teamwork and communication in the operating room by introducing the theatre cap challenge, 2022, van Dalen et al.	Netherlands	(1) To assess whether the name and role instructions as part of the WHO SSC were actually completed; (2) how well the team members were able to remember and recall each other's name; (3) evaluate the introduction of the challenge of the personalised surgical cap in the OR	Observational study	85	OR teams	− Structured debriefing − Visual analog scale (VAS, 0–10)	92% staff approval; ↑ name use	Figure 1: Labeled cap example; Table 3: Team acceptance rates (92%)
What's in a name? Enhancing communication in the operating room with the use of names and roles on surgical caps, 2020, Brodzinskij et al.	USA	Evaluate the impact of personalised surgical caps with the names and roles of the operators on communication in the operating room.	Pilot RCT	129	OB/GYNs, anesthesiologists, nurses, techs	− Observational checklist − Postoperative survey	↑ Name recall (77.8% vs. 55%, *p* = 0.011); ↓ missed communications	Figure 1: Qualitative feedback; Figure 2: Communication metrics;
Personalized scrub caps for identification of surgical trainees, 2023, Agarwal et al.	USA	Investigate if surgical caps for medical residents can help reduce name and role identification errors, microaggressions and delays related to miscommunication in patient care	Cross‐sectional survey	92 (64 completed)	Surgical trainees (53% female)	− Likert‐scale questionnaire (pre/post)	↓ Role misidentification (67.2%); ↓ microaggressions (35.9%, *p* < 0.001)	Figure 1: Cap design; Figure 2A: Misidentification trends; Figure 2B: Patient care errors

### 4.1 Teamwork

Two studies have shown that the use of personalized surgical caps improves teamwork. In fact, the pilot study conducted by Douglas et al. [21] showed a significant improvement in the perception of teamwork, with the median score increasing from 3 to 4 (*U* = 757, *p* < 0.001) by the midwives recruited in the sample; in contrast, physicians, nurses, and technicians showed no significant change in teamwork perception scores before and after surgery. Similarly, a significant improvement in teamwork after the introduction of the personalized surgical caps was found in the study by Wong et al. [27] study: physicians who reported being called by name more often were more likely to perceive an improvement in teamwork (AOR, 3.46; 95% CI, 1.91–6.26; *p*< 0.001) and an improvement in relationships with other team members (AOR, 3.21; 95% CI, 1.76–5.84; *p *< 0.001).

### 4.2 Communication

Communication is the relevant theme of the Brodzinsky et al. [28] RCT: 90.7% of team members wearing the personalized surgical caps reported “easy, barrier‐free” communication compared to 65.0% with caps without first and last name, with a statistically significant difference (95% confidence interval = 79.7%–96.9% for labeled caps vs. 51.6%–76.9% for unlabeled caps, *p* = 0.001). Visual data from primary studies further support these findings. Brodzinsky et al. reported a trend toward increased name use (43 vs. 34 instances) and reduced missed communications (16 vs. 20) during cesarean sections when labeled caps were worn, although these differences did not reach statistical significance. Similarly, Agarwal et al. reported that personalized scrub caps with embroidered names and roles were associated with a 65.6% reduction in name misidentification and a 67.2% reduction in role misidentification among trainees, based on pre–post survey data. Notably, female and URM trainees reported the most significant improvements (e.g., 95% reduction in role misidentification vs. 20.8% in male/non‐URM peers; *p* < 0.001). Although there is a trend favoring more use of names and fewer missed communications with the use of labeled surgical caps, neither group reached statistical significance in Brodzinsky et al.'s study [28]. Missed communications involved different groups of healthcare professionals and different topics, but did not lead to patient damage.

### 4.3 Knowledge of Name and Role

Knowledge of the name and role of each member of the surgical team is reported in four studies included in the systematic review. In the study by Douglas et al. [21] the number of healthcare professionals who reported knowing the names of all staff present in the operating theatre increased after surgery (31% vs. 15%, *χ* ^2^ = 20.2, *p* < 0.001), with the most commonly recalled name being that of the anaesthetist. However, this change was not statistically significant for all professional groups (surgeons or obstetricians, and gynecologists, 59.8% vs. 47.9%, *χ* ^2^ = 4.19, *p* = 0.47; nurses = 55.1% vs. 43.6%, *χ* ^2^ = 3.9, *p* = 0.062). In addition, fewer participants reported knowing all the names of the technicians they worked with in the post‐intervention survey (41% vs. 59%, *χ* ^2^ = 16.6, *p* < 0.001). The study by Wong et al. [27] is also consistent with the data reported by Douglas et al. [21]: medical staff reported a significant improvement in being called by name more often after receiving a labelled surgical cap (86%; 95% CI, 81%–91%; *p* < 0.001). In the Brodzinsky et al. [28] RCT, 77.8% of staff wearing the labelled surgical caps reported knowing the names of all team members when wearing the personalized caps compared to 55.0% wearing the unlabeled caps, a statistically significant difference (95% confidence interval = 64.4%–88.0% for labelled caps vs. 41.6% –67.9%, *p* = 0.011). In the labelled surgical caps group, 92.5% of healthcare workers were aware of the roles of other team members compared to 78.3% who did not wear labelled surgical caps, a statistically significant difference (95% confidence interval = 81.8%–98.0% for labelled caps vs. 65.8%–88.0%, *p* = 0.036). This trend was most evident among obstetricians and anaesthetists. In the pilot study conducted by Agarwal et al. [29], after 6 months of using the personalized surgical cap, 65.6% of OR staff reported that the caps helped reduce name misidentification, 67.2% reported a reduction in role misidentification, and 35.9% reported a reduction in microaggressions. Female and underrepresented minority (URM) residents reported significantly greater improvements in role recognition compared to male/non‐URM peers (95.0% vs. 20.8%; *p* < 0.001). Specifically, 38% of 40 URM residents versus only 5 of 24 non‐URM residents noted reduced misidentification.

## 5 Discussion

The systematic review aims to evaluate the effectiveness of surgical caps personalized with the names and roles of the members of the surgical team to improve teamwork and communication. From the results received in the different studies [21, 27, 28, 29], it appears that the use of personalized or labeled surgical caps promotes better identification among team members and increases their knowledge of colleagues' names and roles, as also reported in the study by Burton et al. [20] The increased frequency of name and role identification results in effective communication and an improved perception of teamwork, as evidenced by the results of some studies included in SR [21, 27, 28, 29]. This is particularly relevant in the hospital setting, where clear identification of team members can promote optimal patient management and greater cohesion within the team. In fact, Lingard et al. [14] found that 30.6% of surgical errors resulted from communication failures, with 20.9% of these being “recipient failures”, such as talking to the wrong person or inappropriate role recognition. Another review conducted on 60 cases of surgical negligence also demonstrated high communication failures, with 73% errors attributed to ambiguity about clear roles and responsibilities [31]. As more and more literature supports the importance of role clarity to reduce surgical errors and improve communication, all team members must engage in optimizing communication. Lack of knowledge of names and roles in the theatre, complex interactions between team members can potentially cause role confusion and unclear expectations during challenging multidisciplinary surgical cases [32]. Furthermore, the SR results indicate that a significant reduction in misidentification is crucial for patient safety and healthcare quality. In addition to the potential safety benefits of knowing the names and ranks of OS colleagues, it is well recognized that the use of personal names and clear role identification have been shown to foster integration, humanize care and improve team bonding [14]. In fact, many successful companies endorse the use of names in communications with and between employees, which is perceived as complimentary and facilitates interpersonal relationships [33]. On the other hand, an impersonal and rigid formality amplifies status asymmetry, negatively influencing cooperation, team goals, and team [33]. Another important observation to come out of this review is the different impact of these strategies among the different subgroups of healthcare professionals: for example, in Douglas et al. [21] study, the name of the first surgeon was the most remembered, while that of the residents was the least remembered, probably due to the continuous turnover of residents in the operating room [7]. In the Agarwal et al. [29] study, female residents and those from underrepresented minorities reported more significant benefits in correctly identifying their name and role than male residents, suggesting that these initiatives could contribute to reducing gender and representation disparities within the healthcare environment. The impact of personalized caps varied across subgroups. Female and underrepresented minority (URM) trainees reported disproportionately greater reductions in name and role misidentification, as well as perceived microaggressions, likely reflecting the influence of pre‐existing biases in the operating room environment, as observed by Agarwal et al. [29]. This aligns with the qualitative findings reported by Brodzinsky et al. [28], in which providers described labelled caps as fostering respect and reducing anxiety during high‐stress situations, especially for professional roles historically prone to depersonalization, such as anesthesiologists and trainees. Medicine has historically been a male‐dominated profession; consequently, residents may be prejudiced by the stereotype that women do not fit the historical image of a doctor [34]. In fact, sex discrimination has been increasingly reported among physicians and other healthcare professionals: in a recent survey of surgeons, 65% of women reported gender discrimination [2]. Other findings from the studies in this SR suggest that personalized surgical headsets contribute to a more professional perception among patients [29]. They also help reduce micro‐aggression when customized headsets are used [29]. Additionally, both staff and patients report greater perceived well‐being when staff wear headsets [28]. These results highlight how tailored surgical headsets can enhance patient confidence and comfort during medical procedures. In summary, the data collected from these studies suggest that the adoption of targeted interventions, such as the use of personalized surgical caps with name and role and other communication improvement strategies, can contribute to improving the quality of healthcare by reducing misidentification errors and promoting a culture of safety and collaboration within healthcare teams.

### 5.1 Limits and Strengths

It is important to acknowledge some limitations of our study. The first limitation of the study is the possible presence of studies in which recruited health care providers did not complete the survey or chose to leave the study in the posttest phase. This may affect the representativeness of the results and potentially lead to an underestimation or overestimation of the effects of the strategies examined. Nonparticipation or dropout of healthcare providers could be due to several factors, including workload, lack of time, or lack of interest in the proposed intervention, and these factors could introduce bias into the review results. Therefore, interpretation of the results was conducted based on the availability and quality of the included data. The strength of this systematic review lies in the originality of the topic, which adds an important perspective to the current clinical practice. This contributes to greater interest in healthcare, as this is a topic on which there is still a significant gap in practical implementation within operating rooms. Furthermore, secondary studies synthesizing existing evidence on this topic have not been identified in the literature, further underscoring the importance and need to conduct a review of the available evidence.

## 6 Conclusion

The systematic review shows that the introduction of surgical caps personalized with the names and roles of them is associated with an improvement in perceived teamwork in the operating room, both in terms of recognition as personal and role identity is fostered, and in terms of communication and better connection between perioperative teams, thus also providing patient safety.

## 7 Implications for Clinical Practice

The use of surgical caps personalized with the names and roles of operating room team members represents a promising clinical practice that merits further investigation and future development. In particular, the implementation of surgical caps in the operating room should involve a larger sample of healthcare professionals. The results of the review provide important insights into future research. It would be useful to examine the effectiveness of surgical cap use in patients undergoing surgery as well, to understand their level of confidence and comfort during medical procedures.

## Author Contributions

**Vincenza Giordano:** conceptualization, supervision, writing – original draft. **Vincenzo Bosco:** writing – review and editing. **Rita Nocerino:** Conceptualization, data curation, visualization. **Dalila De Domenico:** data curation, writing – original draft. **Cristiana Rago:** data curation, supervision, writing – original draft. **Michele Virgolesi:** conceptualization, writing – review and editing. **Teresa Rea:** conceptualization, validation. **Assunta Guillari:** conceptualization, validation.

## Conflicts of Interest

The authors declare no conflicts of interest.

## Transparency Statement

The lead author, Michele Virgolesi, affirms that this manuscript is an honest, accurate, and transparent account of the study being reported; that no important aspects of the study have been omitted; and that any discrepancies from the study as planned (and, if relevant, registered) have been explained.

## Supporting information

PRISMA‐ScR‐Fillable‐Checklist 10Sept2019.

## Data Availability

The data supporting the findings of this study are derived from previously published studies and publicly available data sets. All relevant data used in the systematic review are included within the manuscript and its supporting materials. Further details can be obtained from the corresponding author upon reasonable request.
